# Barriers to tuberculosis and human immunodeficiency virus treatment guidelines adherence among nurses initiating and managing anti-retroviral therapy in KwaZulu-Natal and North West provinces

**DOI:** 10.4102/curationis.v41i1.1808

**Published:** 2018-03-26

**Authors:** Lufuno Makhado, Mashudu Davhana-Maselesele, Jason E. Farley

**Affiliations:** 1 School of Nursing Science, North-West University, South Africa; 2 School of Nursing, Johns Hopkins University, United States

## Abstract

**Background:**

Nurses, as front-line care providers in the South Africa’s health care system, are called upon to deliver integrated interventions for tuberculosis and human immunodeficiency virus (TB and HIV) including nurse-initiated management of anti-retroviral therapy (NIMART) and anti-TB treatment. Adherence to treatment guidelines and factors associated with non-adherence to treatment guidelines among nurses remain under explored.

**Purpose:**

To explore and describe barriers to treatment guidelines adherence among nurses initiating and managing anti-retroviral therapy and anti-TB treatment in KwaZulu-Natal and North West provinces.

**Design:**

This study employed a qualitative exploratory descriptive design.

**Methods:**

Four semi-structured focus group interviews were conducted during 2014 each consisting of four to eight NIMART trained nurses. Audiotaped interviews were transcribed verbatim and analysed using Atlas T.I. software.

**Findings:**

During data analysis, two themes emerged: (1) NIMART trained nurses’ distress about TB and HIV guidelines adherence that is inclusive of lack of agreement with guidelines, poor motivation to implement guidelines, poor clinical support and supervision, resistance to change, insufficient knowledge or lack of awareness and (2) exterior factors inhibiting nurses’ adherence to treatment guidelines which incorporated organisational factors, guidelines-related factors and patient-related factors.

**Conclusion:**

This qualitative study identified that nurses have substantial concerns over guideline adherence. If NIMART trained nurses’ barriers inhibiting adherence to treatment guidelines cannot be remedied, patient outcomes may suffer and South Africa will struggle to meet the 90-90-90 targets.

## Introduction

Adherence to treatment guidelines seems to be a major challenge among nurses caring for people living with human immunodeficiency virus (PLWH) and tuberculosis (TB) patients. Guidelines adherence is also associated with improvement of patient outcomes. Unfortunately, adherence to TB and human immunodeficiency virus (HIV) guidelines among health care providers varies based on services provided as well as with individual perspectives around the stipulated recommendations (Hiransuthikul et al. 2005; Low & Eng 2009; Naidoo et al. 2010; Peterson et al. 2011; Saraceni et al. 2011). The nursing profession, in general, has demonstrated excellent guideline-based care delivery before diagnosis, with initiation of anti-retroviral therapy (ART), as well as patient monitoring (Hiransuthikul et al. 2005; Low & Eng 2009; Naidoo et al. 2010; Peterson et al. 2011; Saraceni et al. 2011). Adherence to treatment guidelines, however, is influenced by many factors, including health care providers’ familiarity with or agreement with guidelines (Crocker et al. 2013) in addition to the time-pressured environment of primary care (Crocker et al. 2013).

Six factors have been consistently reported to influence adherence to treatment guidelines: familiarity, awareness, outcome expectancy, self-efficacy, motivation and agreement (Cabana et al. 1999; Satman et al. 2010; Vashitz et al. 2011). The influence of each of these factors differs by profession (physicians vs. nurses vs. dieticians, for example), educational background, care experience and personality (Satman et al. 2010; Vashitz et al. 2011). Lack of agreement with treatment guidelines, poor outcome expectancy and paucity of evidence supporting the recommendations had been found to be stronger barriers to physicians, whereas practical concerns, such as expertise, workload and patient comfort, were deemed to be more important for nurses (Cabana et al. 1999; Satman et al. 2010; Vashitz et al. 2011).

Nurses as the front-line providers in South Africa’s health care system are essential to integrated interventions for TB/HIV. Factors inhibiting nurse-initiated management of anti-retroviral therapy (NIMART) nurses’ adherence to treatment guidelines in South Africa remain under explored. It is of paramount importance to investigate the relationship between provider adherence to clinical guidelines and its influence towards quality care provision. This study sought to explore and describe barriers to treatment guidelines adherence among nurses initiating and managing ART and anti-TB treatment in Kwazulu-Natal (KZN) and North West (NW) provinces.

## Methods

A qualitative, exploratory descriptive design was used to explore and describe barriers to treatment guidelines adherence among nurses initiating and managing ART and anti-TB treatment in KZN and NW provinces.

### Context of the study

The study was conducted in two districts within two provinces, Ugu district (Kwazulu-Natal Province) and Ngaka Modiri Molema district (North West Province), of South Africa.

### Sampling criteria

Nurse-initiated management of ART-trained nurses initiating and managing ART and anti-TB treatment were sampled using purposive sampling and recruited from the primary health care (PHC) and community health centres (CHC) in KZN and NW provinces which were sampled using systematic random sampling. Sixteen facilities (eight CHCs and eight PHCs) were selected from KZN and NW provinces. Nurses were purposively selected if they were NIMART trained, currently initiating and managing ART for at least 12 months in a CHC or PHC and accredited to provide ART services. The study sample consisted of 24 participants consisting of 4–8 NIMART trained nurses who agreed and consented to participate in the study. All participants were NIMART trained nurses, of whom 17 were female (70.8%). Other demographic features are presented in Table 1.

**TABLE 1:** Participant characteristics.

Characteristics of NIMART nurse	*N*
**Gender**	
Male	7
Female	17
**Age**	24–58
**Experience providing TB and HIV services after NIMART training**	
3 years	13
4 years	11
**Facility type**	
Primary health care clinic (PHC)	11
Community health care centre (CHC)	13

NIMART, nurse-initiated management of anti-retroviral therapy; TB, Tuberculosis; HIV, human immunodeficiency virus.

### Data collection

Data were collected from February to April 2014. Each interview was initiated with the central question ‘what are the barriers to treatment guidelines adherence among NIMART trained nurses’. The central question was discussed in all sessions followed up by probing and follow-up questions in relation to adherence to treatment guidelines. The time of day was selected by the participants prior to the focus group sessions. The focus group interview sessions were carried out in a quiet room at one of the hospitals in Port Shepstone and at North-West University School of Nursing Sciences boardroom, which lasted 90–140 min. All focus group discussions (FGD) were recorded using a digital recorder and were transcribed verbatim for later analysis. Data saturation was reached within the fourth session as no other new information arose from the FGD from both districts.

### Data analysis

Transcripts were compared with audiotapes and field notes to confirm the correctness of the transcribed data. Demographic characteristics were analysed from the focus group demographic data sheet. This study used ATLAS T.I. software program (version 7.0) and followed the basic steps of notice-collect-think (NCT) analysis (Friese 2012). These basic steps enabled the researcher to work in a systematic manner instead of declaring the software to be the method itself (Friese 2012). The researcher started by noticing aspects of the data that led to an idea for a label and began to collect what was noticed in the form of codes (Friese 2012). This involved the following steps: familiarisation with raw data; identifying an index of all the codes and categories to be used from the raw data; applying the index to all the raw data by noting transcripts with the codes; charting all the raw data from the same code in a particular document; and interpreting themes from the charts in relations to the range and strength of opinions, as well as any associations or relationships between themes (Friese 2012). Quotations from these interviews are used in this article to illustrate vital points which had been controlled using available literature.

### Measures to ensure trustworthiness

In order to provide trustworthiness of the qualitative analysis, the researcher followed Guba’s (1981) criteria of ensuring credibility, transferability, dependability and confirmability. Credibility (internal validity) was addressed by having two researchers independently reading and coding the transcribed focus group sessions. Transferability (external validity and generalisability) was enforced by providing rich, thick description (Lincoln & Guba 1985) and sharing the results with content experts and conducting further literature review. Confirmability (objectivity) was assessed by comparing the transcribed focus group sessions with the extensive notes taken by the non-participating note-taker from the focus group. Dependability (reliability) was achieved by triangulation of methods which involved the use of FGD, field notes and observations (Lincoln & Guba 1985).

### Ethical considerations

Ethical clearance for the study was given by the North-West University ethics committee (NWU-000033-13-A9). Provincial Department of Health for NW and KZN granted permission to conduct the study. Voluntary participation and written consent of all participants was sought, and detailed information about the research was provided.

## Results and discussion

Four focus group interview sessions were conducted consisting of 4–8 NIMART trained nurses in each group, two sessions in each province. In the process of empowering nurses in the initiation and management of ART in South Africa, usage and adherence to treatment guidelines seem to be hindered by multifaceted factors. Nurse-initiated management of ART-trained nurses expressed barriers that were inhibiting them from fully adhering to treatment guidelines in their facilities and throughout their caregiving role. Analysis of the interview data exposed two major themes: (1) NIMART trained nurses’ negative distress about TB/HIV guidelines and (2) exterior factors inhibiting NIMART trained nurses’ adherence to treatment guidelines. These themes and associated subthemes are described in Table 2.

**TABLE 2:** Overall themes and subthemes that emerged from the data analysis.

Theme	Subthemes
NIMART trained nurses’ distress towards TB and HIV guidelines adherence	Lack of agreement with the guidelines
	Poor motivation, support & supervision of NIMART trained nurses
	Resistance to change
	Insufficient knowledge and lack of awareness
Exterior factors inhibiting NIMART trained nurses’ adherence to guidelines	Organisational factors
	Guideline recommendation factors
	Patient related factors

NIMART, nurse-initiated management of anti-retroviral therapy; TB, Tuberculosis; HIV, human immunodeficiency virus.

### Theme 1: Nurse-initiated management of anti-retroviral therapy-trained nurses’ distress about tuberculosis/human immunodeficiency virus guidelines adherence

In this study, ‘distress’ refers to the way in which nurses feel, worry and are concerned about using and adhering to the TB/HIV treatment guidelines. The following barriers were identified as leading to their distress, namely lack of agreement with guidelines, poor motivation to implement guidelines, poor clinical support and supervision, resistance to change and insufficient knowledge or lack of awareness.

#### Lack of agreement with the guidelines

The NIMART trained nurses verbalised that the guidelines were not user friendly, indicating the issue of the complexity of the treatment guidelines and stating that they find it hard to work with the TB and HIV guidelines. TB, HIV and Practical Approach to Lung Health and HIV/AIDS in South Africa (PALSA) Plus guidelines were also said to contradict one other. The NIMART trained nurses expressed that:

‘It is not easy to use the guidelines that you don’t understand and again the guidelines have other things that are confusing to me and no one seems to have answers to my queries as well.’ (P9S4, male, 44 years old)

And

‘The guidelines are contradictory, and we (are) dealing with integrated services (TB and HIV) so the TB guideline says this and the HIV or ART guideline says that whereas the PALSA Plus [*another type of clinical guide*] is saying this on the other side.’ (P9S4, male, 44 years old)

Furthermore, NIMART trained nurses also felt that they are not involved in the development of guidelines, or consulted for review and, as a result, they are required to implement the guidelines recommendations as they are regardless of whether they agree or not. Nurse-initiated management of ART-trained nurses highlighted that:

‘I have a problem with the guidelines because I was not involved in the development of it, I don’t know about you nurses but I do have a problem. We are not engaged in the development but are fully expected to implement it whether we agree or not. And In addition, we don’t even get to be consulted before hand if the guideline is suitable for use or not. It’s an obligation and it’s not easy to adhere to something you do not agree to.’ (P12S4, male, 42 years old)

The use of treatment guidelines is discouraged when the NIMART trained nurses perceives it to be unusable in daily practice (Abrahamson, Fox & Doebbeling 2012). Other evidence pointed out that nurses felt the treatment guidelines were too complicated and criticised the treatment guidelines as too ‘cook book, too time consuming and too cumbersome’ (Afreen & Rahman 2014; Christakis & Rivara 1998). However, NIMART trained nurses would be in a better position to follow guidelines that have been developed with nursing input and this may result in a greater sense of ownership. This notion is supported by a study indicating that treatment guideline adherence was high among Dutch general practitioners (GPs) because the guideline was developed by their fellow GPs (Lugtenberg et al. 2011).

#### Poor motivation, support and supervision of nurse-initiated management of anti-retroviral therapy-trained nurses

Lack of support from the managers and programme coordinators was reported to affect NIMART trained nurses’ adherence to treatment guidelines. Nurse-initiated management of ART-trained nurses revealed that:

‘The [*TB & HIV*] programme managers took long to come back to us, they don’t even visit for support unless there is some provincial visit, that’s when they will come and you will find that all the wrongs would have been made right earlier if they took support visits seriously and we wouldn’t have been stuck with the old guidelines or protocols that we using even now.’ (P7S4, female, 46 years old)

And

‘We are not supported or motivated to use the guidelines. We do have programme coordinators, managers and supervisors but they are not supportive or encouraging to us.’ (P5S3, female, 58 years old)

It was evident that NIMART trained nurses feel that the importance of support for treatment guideline use is somewhat ignored and it makes clinical and monitoring and evaluation visits impossible as it was felt that the programme managers and physician mentors do not see the need to provide routine support. Abrahamson et al. (2012) further emphasised that nurses acknowledge the importance of receiving support from physicians in terms of treatment guideline use and this was found lacking in their facilities. In addition, the lack of support further discourages the spirit of cooperation.

Adherence to treatment guidelines was said to be discouraged by the absence of cooperation among health care providers. Nurse-initiated management of ART-trained nurses are not the only health care providers that patients come across and it takes the whole multidisciplinary team to adhere to the treatment guidelines and record everything in the patient files. One NIMART trained nurse articulated that:

‘Most of the trained nurses do not give feedback to other colleagues hence a problem of owning a programme. Nurses need to know that it is not their programme, we work together trained or not trained for one reason only that is provision of quality care and treatment to our patients.’ (P10S2, male, 34 years old)

Another participant emphasised that:

‘We would love to [*sic*] … for our doctors to attend these workshops because you find that our doctors are not, they don’t know any guidelines. It even amazes me in ART some doctors don’t know anything that needs to happen during initiation and as a nurse you will be referring clients and you will be afraid to tell the doctor to say please do one, two, three and this as it will be like you are undermining or what so ever.’ (P4S3, male, 27 years old)

And

‘Some of the challenges that we face are due to lack of teamwork between enrolled nurses, doctors, ENAs [*Enrolled Nursing Auxiliary*] and us as professional nurses, there is no communication between this categories. And we are supposed to work hand in glove with one another for the provision of quality care to our patients.’ (P7S4, female, 46 years old)

This is a major challenge leading to poor cooperation between health care providers. The use of guidelines is discouraged when there is no lateral cooperation between health care workers (Abrahamson et al. 2012; Lugtenberg et al. 2009). It was further emphasised that communication gap is a reason for non-adherence to treatment guidelines and use (Afreen & Rahman 2014). Likewise, nurses work closely with doctors; however, this relationship has had a long history of conflict and often it is stereotyped as ‘us versus them’, that is, nurses versus doctors and vice versa (Lugtenberg et al. 2009). This had been regarded as a very unhealthy approach to teamwork, proper communication and health relationship (Lugtenberg et al. 2009).

#### Resistance to change

Nurse-initiated management of ART-trained nurses expressed that they experience difficulty in coping with the amount of change in the health care system and they find it hard to move from previous ways of doing things as treatment guidelines keep changing and this is because change is difficult for them. One NIMART trained nurse stated that:

‘I think it is very difficult to change, it is very hard to move from the previous practice and the way we use to do things to this NIMART and TB/HIV integrated services. So I think change is a huge problem that needs attention.’ (P9S4, male, 44 years old)

These statements are in line with available literature revealing that health care providers feel that they are not sufficiently motivated to change and that it is hard for them to overcome inertia of what they used to practise versus what they are expected to implement and this is because of habits and routines rooted to them in accordance with previous guidelines (Lugtenberg et al. 2009, 2011). Hence, they felt that this needs to be attended to in order to assist them to cope with the challenges they face and to be motivated efficiently in their care giving role.

#### Insufficient knowledge and lack of awareness

It is of paramount importance that all NIMART trained and untrained nurses receive the necessary orientation as to make them aware of the available treatment guidelines as well as provision of follow-up or in-service training. Most NIMART trained nurses verbalised that they lack necessary education and training needed to keep them updated with the NIMART program and anti-TB treatment to TB and HIV patients. The nurses had this to say about education and training:

‘Lack of training within the facilities is a major challenge in my opinion. Like for instance there is only one NIMART trained nurse in my facility, ‘me’ and what happens like now when I am not there in the facility?’ (P8S2, female, 41 years old)

Another participant added that:

‘Indeed it is a problem and puts a lot of pressure to us as NIMART trained nurses as majority of other nurses are not NIMART trained and it also boils back to the patient and this reduces the adherence to the guidelines as I don’t own the patient.’ (P10S2, male, 34 years old)

Another NIMART trained nurse emphasised that:

‘Again if you not NIMART trained you don’t feel confident to carry out duties of ART initiation and management like us who are trained. Hence some of the patients will end up being attended by a professional nurse who is not trained in NIMART.’ (P10S2, male, 34 years old)

These sentiments are in agreement with previous assertions, namely nurses perceived that a lack of education and training on treatment guidelines can adversely affect the adherence to and use of treatment guidelines (Abrahamson et al. 2012; Theodorou et al. 2012). According to literature appropriate nursing education, in the practical implementation of the guidelines, plays a major role in the care of patients (Abrahamson et al. 2012; Lugtenberg et al. 2009).

Lack of knowledge and awareness with regard to treatment guidelines was said to be a huge challenge. Training and orientation with regard to treatment guidelines should be improved and this should be conducted in their facilities as it reduces other cost and staff shortage implications. Furthermore, the inclusion of treatment guidelines in undergraduate, postgraduate and continuing nursing education can increase the level of awareness and knowledge and thus improve the level of adherence to the treatment guidelines. This supports McCarthy et al. (2013) on the emphasis that the NIMART program and its guidelines can be integrated into nursing education and further into the available national regulatory frameworks so as to ensure nurses’ achievements of the desired outcome, adherence to treatment guidelines, and reach the goal of NIMART program. In addition, this integration of the NIMART in nursing education will be of paramount importance as nurses in South Africa were not trained to deliver NIMART through pre-service education (Zuber et al. 2014) and only received training during their post basic education. Similarly, NIMART training integrated into Community Nursing Science program is a feasible option to increase graduates’ abilities to provide care to PLWH (Farley et al. 2016). This is possible through the encouragement of curriculum innovations that can facilitate the integration of community nursing science and HIV treatment and care (Farley et al. 2016).

### Theme 2: Exterior factors inhibiting nurses’ adherence to treatment guidelines

Exterior factors are those factors inhibiting nurses from adhering to treatment guidelines that NIMART trained nurses themselves have no control over. These factors affect them mentally, physically, emotionally, and socially and have a negative impact on their level of adherence to treatment guidelines. Exterior factors inhibiting nurses’ adherence to treatment guidelines were sub-categorised into organisational, guideline recommendation and patient factors.

#### Organisational factors

Organisational factors included all factors that were related to facilities that were used by participants rendering TB/HIV services to clients and patients. These factors include time pressure, shortage of staff, heavy workload, poor communication, organisational constraints, guideline availability, poor guideline accessibility and provider accountability and overall organisational environment, and these factors were presented on both facilitators and barriers to adherence to treatment guidelines. Nurses trained in NIMART indicated that they do not have time to utilise the treatment guidelines in the facility and this is normally because of the quality assurance time frames set forth by the Department of Health to monitor patient stay in the facility. One NIMART trained nurse articulated that:

‘The issue of workload and timeframe [*Time allocated for each patient to spend from arrival until patient leaves the facility*] of the patients as stipulated by the quality assurance is a big challenge to guidelines adherence and usage in my clinic.’ (P11S4, female, 33 years old)

Another participant added:

Workload is a major challenge when it comes to proper adherence to treatment guidelines, we don’t have enough time and we don’t have enough time to use the guidelines; hence the workload prevents us from adhering to treatment guidelines.’ (P3S1, female, 26 years old)

The ability to use the guidelines and adhere to them is basically impeded by lack of time (Abrahamson et al. 2012; Lugtenberg et al. 2009). Heavy workload had been verbalised to prevent and deprive NIMART trained nurses from using and adhering to treatment guidelines as there is much more to do than look at what the guidelines are saying.

The other factor that was revealed was shortage of staff. Nurse-initiated management of ART-trained nurses indicated that:

‘The shortage of staff … you find that it’s not everybody that is trained on that particular subject. But now, in addition to this shortage of staff, you find that the person who is not trained doesn’t know how the guideline of that particular subject look like, he or she has never seen such guideline and he or she has to work in that particular area. For instance, you find that the person had never been NIMART trained and never been exposed to ART initiation but have to initiate ART because he/she is the next available nurse and there is no one to help the patient, the patient cannot be denied access to health care just because the trained nurse is not available, the patient has nothing to do with that.’ (P6S1, male, 26 years old)

Another participant indicated:

‘Whether the NIMART trained nurse is on leave, off duty or sick, TB/HIV service needs to be provided and it is not the NIMART trained nurse alone not adhering to guidelines but also other nurses that provide the same services in the absence of the NIMART trained nurse.’ (P4S1, female, 56 years old)

Shortage of nurses presents a major challenge in the provision of care to TB/HIV patients as this affects nurses going off duty, attending training, meetings, annual leave, maternity and sick leave, and these create challenges as patients will be attended to by any available nurse on duty. This affects the level of adherence in the patients’ records as even the health care providers who are not trained are involved in the management of TB/HIV because of shortage of trained nurses.

#### Guidelines-related factors

Treatment guidelines unavailability is another challenge faced by NIMART trained nurses, which leads to poor adherence. One participant indicated that:

‘Some of our clinics don’t even have the so-called treatment guidelines and this truly affects the level of adherence and guideline use.’ (P6S3, female, 49 years old)

It was evident that some facilities have one or no copy of the treatment guidelines, and this presents a problem when one needs to refer to a particular program on how to go about TB/HIV services. Another aspect is that some facilities have TB/HIV treatment guidelines, but they are not accessible to the NIMART nurses for use. Poor communication between NIMART nurses and other health care providers as well as the administrative and managerial staff was noted. One participant expressed the following sentiment:

‘When new things come from the government like guidelines it doesn’t even go to the media to tell people that there is this change. We are the last people to hear that there is this change and it needs to be implemented [*sic*], we haven’t heard of it yet, not ready yet so I become very offended as I am expected to implement that which is not communicated to me. It’s more of politics of our government and nothing else.’ (P4S3, male, 27 years old)

This was consistent with a recent study emphasising that the quality and quantity of communication received regarding the use of treatment guidelines grossly and adversely influence the usage of such guidelines (Abrahamson et al. 2012).

Nurses reported that guidelines were not clear and not easy to use and very complex for them. Nurse-initiated management of ART-trained nurses verbalised that a simplified and user friendly version of the guidelines can promote guidelines use and adherence thereto, for example:

‘Guidelines are not clear and easy to use they are too complex for me. Why can’t they simplify them and make them user friendly. The problem is not adherence but use. You can only adhere to something that you are using.’ (P11S4, female, 33 years old)

Another NIMART trained nurse added:

‘I agree we have to use the guidelines to adhere to it. However, majority of you will agree with me and the past speaker that we normally don’t find guidelines user friendly.’ (P12S4, male, 42 years old)

This was also consistent with the findings of Lugtenberg et al. (2009) revealing that guidelines recommendations were found to be unclear or confusing or too complex or not easy to use in practice. Treatment guidelines are supposed to be easy to use and clear, and the way to resolving this is by offering our NIMART trained nurses a simplified and user friendly treatment guideline. A clear and simple guideline recommendation can address the complexity of the guidelines, and this can be presented in multiple versions, that is, algorithms, one- or two-page summaries or web-based versions with hyperlinks to more detailed information (Lugtenberg et al. 2009). This will reduce time NIMART trained nurses take to go through the guidelines as it will be easy for them to browse through a simplified treatment guideline.

#### Patient related factors

Patients also have a role to play in the level of adherence. One NIMART trained nurse indicated this:

‘Like in my clinic, the patients come early and if you delay them they will shout at you and report you, so we don’t have time since we have to prioritise the patients before whatever we have to do.’ (P4S1, female, 56 years old)

And the NIMART trained nurses further emphasised that:

‘Maybe the government should give the clients or patients to decide if they want to start ART voluntarily before meeting the eligibility criteria, so that it may also reduce defaulters as they take charge of their health and treatment.’ (P1S1, female, 43 years old)

Nurse-initiated management of ART-trained nurses felt that they themselves sometimes do not adhere to treatment guidelines because if they delayed, patients become angry and for this reason the nurse feels that it is important to prioritise and provide health care services within the time frame given rather than exceeding it while looking for and adhering to all guideline requirements.

The study was conducted in only two different contexts and purposive sampling was used; thus, the study cannot be generalised to other contexts regardless of its contribution. It is also suggested that this study can be the basis of evaluating these barriers in the wider participation quantitatively.

## Implications and conclusions

This study identifies themes regarding adherence to HIV and TB treatment guidelines in two provinces of South Africa. Identifying, acknowledging and addressing barriers to treatment adherence are essential, as they have a potential to jeopardise the delicate progress in the provision of TB/HIV services in the country. Although it is clear that nurses have played an extraordinary role in the increase in the number of PLWH initiated on ART [Global Health Observatory (GHO) data 2016], challenges still exist and must be explored. The UNAIDS 90-90-90 strategy (UNAIDS 2014) is a simplified approach to targets for HIV care. Although these targets are clear, they are not achievable without the support of front-line care providers, particularly nurses. As noted in this study, nurses are calling out for greater support to improve guideline adherence in HIV care as well as TB/HIV co-infection. Large HIV nursing groups have called for greater support for nurses as front-line care providers and growing support has been noted, as seen in the 2016 International AIDS Conference in Durban, South Africa (Farley 2016).

The findings from this study demonstrate that adherence to TB/HIV treatment guidelines is a multifaceted issue and innovative solutions must be designed to address these barriers. There is clearly a need to increase communication and supportive supervision between clinical and facility management with NIMART nurses. Human and clinic-level resources are required to reduce the workload. Efficiencies and alignment of required clinical documentation for co-infected patients are essential. For example, the use of one patient record, register and patient documentation card for TB/HIV services would reduce the volume of required documentation and allow greater focus on patient care services.

Nurse-initiated management of ART nurses believed they are unsupported. Greater involvement of mentors will increase guided and supported TB/HIV services provision. Simple, clear and understandable treatment guidelines that are available in all consulting rooms will decrease time spent looking for treatment guidelines and improve efficiency of services. It is imperative that nursing representation is included on guideline development committees as well as decision-making bodies. This will increase nursing buy-in and support for clinical guidelines as well as ensure the guidelines are developed with the lens of the front-line health care provider.

The inclusion of treatment guidelines in undergraduate and postgraduate education and continuing nursing education can increase the level of awareness and knowledge and thus improve the level of adherence to the treatment guidelines. Relevant updates and follow-up training should be provided to advance their skills, proficiency, knowledge and attitudes towards TB/HIV treatment guidelines with the use of continuous workshops, conferences, in-service and follow-up training programmes. This should be done through continuous professional development (CPD) programmes that the South African Nursing Council (SANC) should reinforce especially during renewal of the licencing certificate on a yearly basis. However, emphasis on how to use the guidelines in clinical practice needs to be promoted and applied.

In conclusion, this study explores and describes barriers to treatment guidelines adherence among NIMART trained nurses initiating and managing ART and anti-TB treatment. Findings revealed that barriers were inclusive of NIMART trained nurses’ negative attitudes towards TB/HIV guidelines and other exterior factors inhibiting nurses’ adherence to treatment guidelines. Nurse-initiated management of ART-trained nurses’ negative attitudes towards TB/HIV guidelines entailed lack of agreement with the guideline, poor motivation, support and supervision, resistance to change and insufficient knowledge, whereas exterior factors inhibiting nurses’ adherence to treatment guidelines comprised organisational, guideline recommendation and patient factors. If NIMART trained nurses’ barriers inhibiting adherence to treatment guidelines cannot be remedied, the country of South Africa will struggle to meet the 90-90-90 targets.
